# Cardiac papillary fibroelastoma: an unusual cause of hemichorea secondary to tumour embolism

**DOI:** 10.1093/ehjcr/ytac035

**Published:** 2022-02-03

**Authors:** Teruyoshi Uetani, Shinji Inaba, Yuki Yamanishi, Riko Kitazawa, Osamu Yamaguchi

**Affiliations:** 1 Department of Cardiology, Pulmonology, Hypertension and Nephrology, Ehime University Graduate School of Medicine, Toon, Ehime 791-0295, Japan; 2 Department of Neurology and Clinical Pharmacology, Ehime University Graduate School of Medicine, Toon, Ehime 791-0295, Japan; 3 Department of Molecular Pathology, Ehime University Graduate School of Medicine, Toon, Ehime 791-0295, Japan

A 74-year-old man presented to our hospital with acute onset of right hemichorea. He had no medical history of hereditary disease, inflammatory, or endocrine disorders that could be associated with involuntary movement. Although the patient had no clinical findings suggestive of infective endocarditis, it was not possible to accurately differentiate between tumour, thrombus, and sub-clinical endocarditis. Brain diffusion-weighted magnetic resonance imaging (MRI) revealed multiple cerebellar infarctions (*Panel A*) while brain MRI showed no obvious abnormalities in the basal ganglia region. Contrast-enhanced computed tomography showed renal and splenic infarction (*Panel B*). Moreover, a transoesophageal echocardiogram showed a mobile mass attached to the aortic valve moving back and forth between the ascending aorta and the left ventricular outflow tract (*Panel C, Video 1*). Based on these findings, we concluded that ischaemic stroke due to cardiac mass was the underlying cause of the hemichorea in this patient. During the hospitalization, the patient had an exacerbation of involuntary movements. Repeat echocardiography revealed the reduction in the size of the mass and brain MRI showed acute multiple cerebral infarctions. Therefore, we decided to surgically remove the residual mass to prevent further embolic events. Histopathological examinations with haematoxylin–eosin staining revealed papillary fibroelastoma (*Panel D*), a rare primary cardiac tumour, with organized thrombus (*Panel E*). After the surgery, we started anticoagulation with warfarin for the prevention of thrombotic event. His hemichorea disappeared 6 months after the onset of symptoms.

**Figure:**
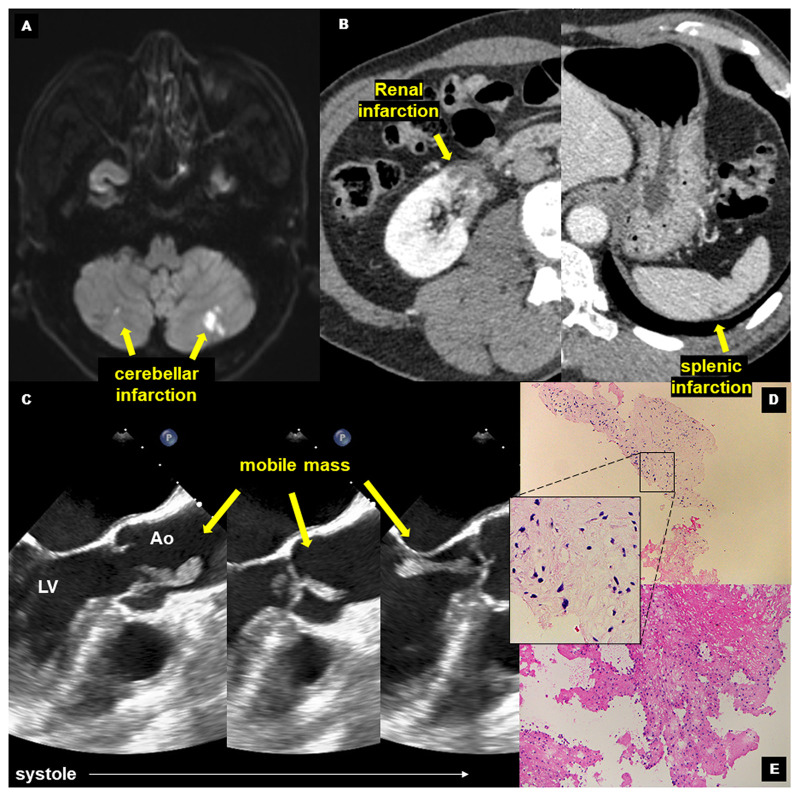


Ischaemic stroke due to embolization of cardiac papillary fibroatheroma is well known. However, hemichorea secondary to tumour embolism is a rare presentation, especially in adults without valve dysfunction. This case study describes an unusual clinical presentation of hemichorea due to cardiac papillary fibroelastoma.

(*Panel A*) Brain magnetic resonance imaging showing cerebral infarction. (*Panel B*) Contrast-enhanced computed tomography showing renal and splenic infarction. (*Panel C*) Echocardiogram showing a mobile mass attached to the aortic valve. (*Panels D* and *E*) haematoxylin–eosin staining showing papillary fibroelastoma (*Panel D*) and organized thrombus (*Panel E*).

**Consent:** The authors confirm that written consent for submission and publication of this case report including images and associated text has been obtained from the patient in line with COPE guidance.

